# Randomized clinical trial comparing octreotide and scopolamine butylbromide in symptom control of patients with inoperable bowel obstruction due to advanced ovarian cancer

**DOI:** 10.1186/s12957-015-0455-3

**Published:** 2015-02-15

**Authors:** Xingang Peng, Peige Wang, Shikuan Li, Guangyong Zhang, Sanyuan Hu

**Affiliations:** Department of general surgery, QiLu hospital of Shandong University, Jinan, 250012 PR China; Department of Emergency General Surgery, The affiliated hospital of Qingdao University, Qingdao, 266003 PR China

**Keywords:** Bowel obstruction, Ovarian cancer, Octreotide, Scopolamine butylbromide

## Abstract

**Background:**

The aim of this randomized controlled study was to determine whether octreotide (OCT) or scopolamine butylbromide (SB) was the more effective antisecretive drug controlling gastrointestinal (GI) symptoms due to malignant bowel obstruction (MBO) caused by advanced ovarian cancer.

**Methods:**

Ninety-seven advanced ovarian cancer patients with inoperable MBO were randomized to OCT 0.3 mg/day (OCT group, *n* = 48) or SB 60 mg/day (SB group, *n* = 49) for 3 days through a continuous subcutaneous infusion. The following parameters were measured: episodes of vomiting, nausea, dry mouth, drowsiness, and continuous and colicky pain, using a Likert scale corresponding to a numerical value (none 0, slight 1, moderate 2, severe 3) recorded before starting the treatment (T0) and 24 h (T1), 48 h (T2), and 72 h after (T3) and the daily quantity of GI secretions through the Nasogastric tube (NGT) during the period of study. One patient in the SB group is not included in any assessments since she withdrew consent prior to receiving any treatment because of rapidly progressing cancer.

**Results:**

OCT significantly reduced the amount of GI secretions at T1, T2, and T3 (*P* < 0.05) compared with SB. NGT secretions significantly reduced at T1, T2, and T3 compared with T0 (*P* < 0.05) in the OCT group, while in the SB group, only at T3, NGT secretions significantly reduced compared with T0. OCT treatment induced a significantly rapid reduction in the number of daily episodes of vomiting and intensity of nausea compared with SB treatment. No significant changes were observed in dry mouth, drowsiness, and colicky pain after either drug. Continuous pain values were significantly lower in the OCT group than in the SB group at T2 and T3 (*P* < 0.05).

**Conclusions:**

At the doses used in this study, OCT was more effective than SB in controlling gastrointestinal symptoms of bowel obstruction. Further studies are necessary to understand the role of hydration more clearly in such a clinical situation.

## Background

Ovarian cancer is the sixth most common cancer among women worldwide [1]. As the surgical treatment of gynecologic cancer becomes more sophisticated and chemotherapeutic approaches become more effective, patients’ prognoses have improved with respect to the duration of their disease-free state. However, when cancer does recur and effective treatment options have been exhausted, the focus of management shifts from curative intent to palliation of symptom manifestations and complications [2]. Malignant bowel obstruction (MBO) is the most common and distressing complication in patients with advanced or recurrent ovarian cancer with an incidence of about 15%–35% [3,4].

Etiologies of MBO in ovarian cancer include extrinsic occlusion of the lumen, malignant infiltration of bowel muscles or nerves, carcinomatosis with involvement of mesentery, and abdominal or pelvic postoperative or tumoral adhesions [4]. Secondary intestinal immobility resulting from opioids and certain antiemetics also plays a role in MBO [3]. MBO results in an accumulation of gastric, pancreatic, and biliary secretions; reduced absorption of water and sodium; and an increase in water and sodium secretion because of increased gastric distension. This pathophysiology results in vomiting, nausea, pain, esophagitis, constipation, and/or diarrhea. Once bowel obstruction has occurred, the median life expectancy drops to approximately 4 months [5]. Although these patients’ survival time may be short, their suffering remains great. Understanding and treating the signs and symptoms of MBO in ovarian cancer patients require urgent attention.

Many patients are unfit for surgery or further chemotherapy and radiotherapy due to the presence of diffuse intraperitoneal carcinomatosis, multiple partial bowel obstruction points, ascites, and/or previous radiotherapy, or because of their poor overall functional status. The aim of any further treatment is to relieve the symptoms related to bowel obstruction and improve the quality of life (QoL). Conservative treatment of inoperable MBO has been found to be effective in controlling the distressing symptoms [6]. In particular, the administration of nasogastric tube (NGT) and liquid supplementation has proved to be effective in controlling gastrointestinal symptoms caused by bowel obstruction [6]. However, prolonged NGT is not recommended because of common complications, such as mucosal erosion and hemorrhage, esophagitis, and aspiration pneumonia, which seriously affect QoL in inoperable MBO patients [7].

Drug treatment without NGT, such as analgesics, antiemetics, and antisecretory drugs, is successful in most patients [8]. To reduce gastrointestinal (GI) secretions, two classes of antisecretory drugs are used: anticholinergics, such as scopolamine butylbromide (SB), and somatostatin (SMS) analogs, such as octreotide (OCT). The two drugs have different mechanisms. The anticholinergic activity of SB decreases the tonus and peristalsis in smooth muscle, both by competitive inhibition of muscarinic receptors at the smooth muscle level and by impairment of ganglionic neural transmission in the bowel wall [9,10]. SB can also reduce intestinal secretions because of muscarinic cholinergic receptors on mucosal cells of the intestinal lumen and in human salivary glands [9,10]. OCT, as an analog of SMS, modulates gastrointestinal function by reducing gastric acid secretion, slowing intestinal motility, decreasing bile flow, increasing mucous production, and reducing splanchnic blood flow [11,12].

However, there have been no comparative studies concerning the efficacy and safety of SB and OCT in reducing symptoms and gastrointestinal secretions in bowel obstructed patients with advanced ovarian cancer in China. Accordingly, we conducted the first clinical study to compare the efficacy of SB and OCT for the control of nausea/vomiting in patients with MBO who were unlikely to respond to any other therapy.

## Methods

### Patients

This study was a prospective clinical trial at Department of general surgery, Qilu Hospital of Shandong University between January 2010 and December 2013. It was approved by the Ethics Committee of Qilu Hospital. All study participants provided written informed consent before randomization.

Inclusion criteria included a diagnosis of documented recurrence of ovarian cancer and the presence of a bowel obstruction based on a compilation of clinical signs, symptoms, and/or radiographic evidence. Available oncologic therapies for tumor control had been exhausted for these patients. The obstruction could not be surgically correctable, as assessed by the patient’s gynecologic oncology surgeon. Furthermore, patients must have had a life expectancy greater than 2 months and ability to give a written informed consent. The following were excluded: (1) patients having already undergone treatment with the drugs under study; (2) patients with cognitive failure; (3) patients with a current history of diabetes mellitus, pancreatitis, or active biliary disease.

### Treatment

**Figure 1:**
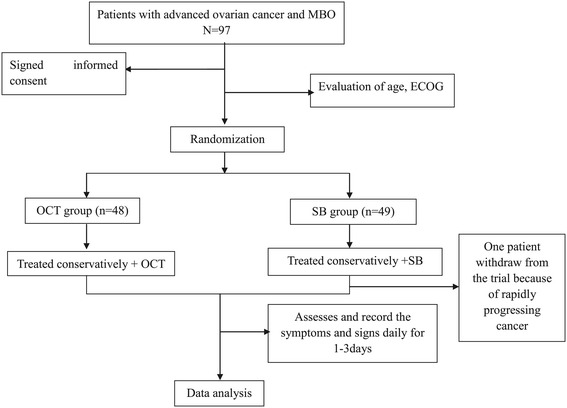
**Trial flow chart.**

After informed consent was obtained, eligible patients were randomized to the OCT group or the SB group. Randomization was achieved by means of computer-generated random numbers. The trial flow chart is showed in Figure 1.

NGT was introduced, and all the patients were managed with intravenous fluids in order to restore the loss of fluid and electrolytic balance. Commonly, as much as 3.5 l of isotonic saline solution was required if the patient shows signs of severe dehydration, but considerably less if such signs were absent. Energy is provided by glucose solutions and vitamins (vitamins B6, C, and K), Coenzyme A and adenosine triphosphate are necessary as supplements as they are essential for the maintenance of normal metabolic function. After the patient has formed adequate urine, potassium chloride would be added to the infusion. Pain therapy was carried out concurrently according to the World Health Organization (WHO) guidelines [13]. From T0 to T3, the patients were not given steroids, antiemetics, anticholinergics, H_2_ blockers, or omeprazole.

Patients in the OCT group and SB group were given OCT (Sandostatin, Novartis Pharma Stein AG) 0.3 mg or SB (Boehringer Ingelheim Pharma GmbH & Co.KG) 60 mg daily by a continuous subcutaneous infusion. These dosages were extrapolated by previous experience [9,14]. If signs of intolerance such as hypotension, hypertension, chest pain, shortness of breath, or anaphylactic reaction occurred, patients were removed from the study and received no further OCT or SB.

### Data collection

Age and performance status according to the ECOG scale were evaluated for all patients. Episodes of vomiting were recorded. The intensity of the following symptoms was evaluated: nausea, dry mouth, drowsiness, and continuous and colicky pain. All symptoms were assessed through a Likert scale corresponding to a numerical value: 0 = none, 1 = slight, 2 = moderate, 3 = severe. All the symptoms were recorded before starting the treatment (T0), 24 h (T1), 48 h (T2), and 72 h (T3). Moreover, we recorded the daily quantity of GI secretions through the NGT.

### Data analysis

Differences between the two treatment groups were assessed using a *χ*^2^ test or Fisher’s exact test for categorical variables. A non-parametric test, the Mann–Whitney *U*-test was used for continuous variables. The Wilcoxon signed ranks test was used to compare the data at different times. A *P* value less than 0.05 was considered statistically significant. Statistical Package for Social Scientists (SPSS, version 18.0, IL) was used for all analyses.

The data were analyzed only for the 3-day study period. After 3 days, researchers were free to change to the alternate drug, to administer SB and OCT in association, to carry on with the same treatment if it proved efficacious, or to increase the dosages of OCT or SB.

## Results

### Patient characteristics

Ninety-seven patients with advanced ovarian cancer and MBO were randomly assigned to the OCT and SB groups. One patient in the SB group is not included in any assessments since she withdrew consent prior to receiving any treatment because of rapidly progressing cancer. Therefore, 96 patients were assessed in the final analysis. There were 48 patients in the OCT group and 48 patients in the SB group. There were no significant differences between the two groups with the characteristics, predominant sites of ovarian cancer as assessed by CT scanning, number of previous operations, and predominant symptoms by patient report (Table 1).

**Table 1:** **Demographic and clinical parameters of patients on admission**

**Characteristic**	**OCT group (** ***n*** **= 48)**	**Control group (** ***n*** **= 48)**	***P***
Age (mean ± S.D., year)	54.2 ± 7.3	53.2 ± 7.9	0.532
ECOG			0.604
3	10	8	
4	38	40	
Predominant sites of cancer (CT scanning)			0.871
Pleural	3	2	
Peritoneal	15	19	
Omental	3	4	
Liver metastasis	7	5	
More than one site	20	18	
Predominant symptoms			0.677
Nausea and vomiting	46	44	
Bloating	20	16	0.399
Constipation	16	19	0.525
Abdominal pain	35	29	0.194

### Treatment outcome

All patients showed well tolerance to OCT or SB. At T0, no significant difference of NGT secretions was showed between the OCT and SB groups (1,515.1 ± 401.2 VS. 1,486.2 ± 432.4 ml, *P* > 0 05, Table 2). At T1, T2, and T3, NGT secretions in the OCT group were significantly less than that in the SB group (T1: 563.6 ± 315.1 VS. 1,206.9 ± 278.2, *P* < 0.05; T2: 355.4 ± 205.4 VS. 808.5 ± 312.6; *P* < 0.05; T3: 298.5 ± 189.2 VS. 783.4 ± 258.6, *P* < 0.05, Table 2). In the OCT group, NGT secretions significantly reduced at T1, T2, and T3 compared with T0 (*P* < 0.05), while in the SB group, only at T3, NGT secretions significantly reduced compared with T0 (Table 2).

**Table 2:** **Daily quantity of GI secretions through the NGT (ml, mean ± SD)**

**NGT secretions**	**OCT group**	**SB group**
T0	1,515.1 ± 401.2	1,486.2 ± 432.4
T1	563.6 ± 315.1^§*^	1,206.9 ± 278.2
T2	355.4 ± 205.4^§*^	808.5 ± 312.6
T3	298.5 ± 189.2^§*^	783.4 ± 258.6^*^

*P < 0.05 for comparison with T0; ^§^ *P* < 0.05 for comparison between groups.

At T1 and T2, the number of episodes of vomiting in the OCT group was significantly less than that in the SB group (*P* < 0.05). Significant reductions in the number of episodes of vomiting were evidenced in the OCT group at T1, T2, and T3 (*P* < 0.05), whereas the reduction was significant only at T3 in the SB group (*P* < 0.05) (Table 3). Significant reduction in the intensity of nausea was reported in the OCT group at T2 and T3 (*P* < 0.05), while no differences were found in the SB group. The intensity of nausea was significantly lower in the OCT group than that in the SB group at T2 and T3 (*P* < 0.05; Table 3). No significant changes were observed in dry mouth, drowsiness, and colicky pain after either drug. Continuous pain values were significantly lower in the OCT group than in the SB group at T2 and T3 (*P* < 0.05, Table 3).

**Table 3:** **Episodes of vomiting, nausea, dry mouth, drowsiness, colicky pain and continuous pain, symptom distress score**

**Symptom**	**Group**	**Point in treatment**			
		**T0**	**T1**	**T2**	**T3**
Vomiting	OCT	5.8 ± 0.6	1.5 ± 0.3^*^	0.5 ± 0.3^*^	1.2 ± 0.5^*^
	SB	5.4 ± 0.8	4.1 ± 0.7^§^	2.3 ± 0.6^§^	2.0 ± 0.8^*^
Nausea	OCT	2.5 ± 0.4	1.5 ± 0.1	0.3 ± 0.3^*^	0.2 ± 0.1^*^
	SB	2.8 ± 0.5	1.3 ± 0.4	1.2 ± 0.4^§^	1.0 ± 0.3^§^
Dry mouth	OCT	2.1 ± 0.1	1.8 ± 0.3	1.9 ± 0.2	1.8 ± 0.1
	SB	1.9 ± 0.2	2.6 ± 0.4	2.7 ± 0.3	2.5 ± 0.4
Drowsiness	OCT	1.6 ± 0.3	1.7 ± 0.2	1.6 ± 0.2	2.0 ± 0.3
	SB	1.7 ± 0.2	1.8 ± 0.1	1.5 ± 0.3	1.7 ± 0.3
Colicky pain	OCT	0.4 ± 0.1	0.3 ± 0.1	0.2 ± 0.2	0.4 ± 0.1
	SB	0.5 ± 0.2	0.4 ± 0.1	0.4 ± 0.3	0.3 ± 0.1
Continuous pain	OCT	1.7 ± 0.2	0.9 ± 0.4	0.5 ± 0.3	0.6 ± 0.3
	SB	1.9 ± 0.1	1.4 ± 0.3	1.3 ± 0.1^§^	0.7 ± 0.1^§^

Data are expressed as mean ± SD.

*P < 0.05 for comparison with T0; ^§^ *P* < 0.05 for comparison between groups.

## Discussion

MBO is a challenging complication of advanced ovarian cancer. Administration of analgesic, antisecretive, and antiemetic drugs has proved to be a valid method of controlling the symptoms of inoperable MBO. Many studies have documented the efficacy of SB and OCT in MBO patients with advanced cancer [7-9,15,16]. The results of our study confirm the capacity of both drugs to reduce GI secretions in patients with inoperable MBO and advanced ovarian cancer. At the doses used in this study, OCT turned out to be more effective than SB in controlling vomiting, although these differences tended to be less pronounced after 3 days. Thus, OCT seems to have a shorter onset of activity than SB, which requires some days before giving a significant effect. About seasickness and continuous pain, we also observed the effect of the OCT is superior to SB probably as the result of a reduction in gastrointestinal secretions and gastric distension, mainly inducing nausea and continuous pain. Previous studies suggest that the principal mechanism of fluid secretion in bowel obstruction depends on vasoactive intestinal peptide (VIP)-induced inflammatory events [17]. OCT has been shown to have a potent anti-VIP effect resulting in the inhibition of intestinal secretion [18].

Parenteral hydration use in the care of terminal cancer patients is still a controversial topic [19]. The main goal of hydration is considered to meet the water/electrolyte baseline requirements and to correct or prevent symptoms related to dehydration, such as thirst, dry mouth, altered mental status, drowsiness, constipation, postural hypotension, and asthenia [20]. However, anecdotal experience has revealed that hydration may serve as a fuel for an obstructed bowel and increase the risk of vomiting [16]. Moreover, Burge et al. [21] showed that patients with advanced cancer receiving less fluid than 750 ml/day do not experience much more thirst than those receiving more than 750 ml/day. Bruera et al. [22] found no significant differences between the hydration group and the placebo group in four dehydration symptoms and concluded that hydration at 1 l per day did not improve symptoms, quality of life, or survival compared with placebo. Further studies are necessary to make advantages and disadvantages of hydration in such circumstances clearer.

Although all patients in our study received enough parenteral hydration, some patients underwent intolerable dry mouth, especially in the SB group. Dry mouth is a subjective feeling of mouth dryness and is not always accompanied by a detectable decrease in salivation [8]. One of the causes of dry mouth is nasal blockage caused by NGT leading to fluid loss from rapid evaporation due to breathing through the mouth. The second reason is reduction of mastication which plays an important role in the regulation of salivary secretion by the effects mediated through somatic afferent nerves of the oral mucosa and in the periodontal tissues. Moreover, SB can produce dry mouth because of the muscarinic receptors in human salivary glands which can explain why patients on SB had a trend toward greater dry mouth intensity.

The study may have some limitations owing to the small number of enrollable patients. Moreover, we designed the trial for a 3-day period only because of the patients’ poor conditions, which could worsen daily and create further problems in determining therapeutic efficacy. Furthermore, our study did not include a crossover study which asks for a washout period between the two different treatments, and this is not recommended in patients with advanced cancer.

## Conclusions

In conclusion, in inoperable MBO, OCT was found to be more effective than SB in relieving gastrointestinal symptoms of advanced ovarian cancer patients. Therefore, OCT should be considered as a first-choice antisecretive drug. Further, well-designed studies are necessary to better evaluate the role of parenteral hydration, not only in symptom control but also regarding a possible influence in increasing GI secretions in patients with inoperable MBO. Moreover, it would be interesting to evaluate the time required to remove the NGT on administering OCT and SB from the beginning of the treatment of MBO patients.
